# Fluoxetine Suppresses Glutamate- and GABA-Mediated Neurotransmission by Altering SNARE Complex

**DOI:** 10.3390/ijms20174247

**Published:** 2019-08-30

**Authors:** Vesna Lazarevic, Ioannis Mantas, Ivana Flais, Per Svenningsson

**Affiliations:** Translational Neuropharmacology, Department of Clinical Neuroscience, Center for Molecular Medicine, Karolinska Institute, 111 22 Stockholm, Sweden

**Keywords:** fluoxetine, presynaptic activity, SNARE complex, PKC, VGCCs

## Abstract

Major depressive disorder is one of the most common neuropsychiatric disorders worldwide. The treatment of choice that shows good efficacy in mood stabilization is based on selective serotonin reuptake inhibitors (SSRIs). Their primary mechanism of action is considered to be the increased synaptic concentration of serotonin through blockade of the serotonin transporter (SERT). In this study, we described an alternative mode of action of fluoxetine (FLX), which is a representative member of the SSRI class of antidepressants. We observed that FLX robustly decreases both glutamatergic and gamma-Aminobutyric acid (GABA)-ergic synaptic release in a SERT-independent manner. Moreover, we showed that this effect may stem from the ability of FLX to change the levels of main components of the SNARE (solubile *N*-ethylmaleimide-sensitive factor attachment protein receptor) complex. Our data suggest that this downregulation of SNARE fusion machinery involves diminished activity of protein kinase C (PKC) due to FLX-induced blockade of P/Q type of voltage-gated calcium channels (VGCCs). Taken together, by virtue of its inhibition at SERT, fluoxetine increases extracellular serotonin levels; however, at the same time, by reducing SNARE complex function, this antidepressant reduces glutamate and GABA release.

## 1. Introduction

Major depressive disorder (MDD) is one of the most common diseases in the world. The lifetime risk of MDD is 20–25% for women and 7–12% for men. It is a leading cause of disability-adjusted life years (DALYs). MDD diagnosis is based on at least one episode of depressed mood, lack of motivation, cognitive dysfunction, and autonomic disturbances; however, the exact pathophysiological aspects of the disease remain largely unknown. The current view on MDD mechanism includes chronic hypothalamic–pituitary axis (HPA) hyperactivity, neuroinflammatory aspects, and neuroplasticity disturbances [1,2,3,4]. In the 1950s, it was discovered that enhancing the monoaminergic neurotransmission has a strong impact on alleviating depressive symptoms [1,5,6]. Moreover, depressive mood traits and MDD are frequent comorbidities of neurodegenerative disorders, especially Parkinson’s disease (PD) [7,8]. Selective serotonin reuptake inhibitors (SSRI) are considered as antidepressants with high combined efficacy and tolerability [9]. For this reason, SSRIs are the most widely prescribed antidepressants in a lot of countries. Two of the most renowned representatives of this antidepressant class are fluoxetine (FLX) and escitalopram [10]. It is believed that their mode of action consists of the blocking the serotonin transporter (SERT). Binding of SSRIs on SERT leads to the accumulation of serotonin in the synaptic cleft and subsequent greater occupation of serotonin receptors (5HTRs). Enhancement of 5HTR activity is interconnected with neuroplasticity changes that lead to upregulation of synaptic proteins and subsequent denser dendritic spines. These synaptic modifications were suggested to involve augmented de novo synthesis and the release of brain-derived neurotrophic factor (BDNF) in the prefrontal cortex (PFC) and hippocampal complex (HPC) [1,4].

There are indications that glutamate has a pivotal role in the pathophysiology of mood disorders like MDD [11,12,13]. Specifically, it is supposed that MDD is characterized by an excess of glutamate exocytosis in the PFC and HPC [12]. The major evidence that supports the aforementioned hypothesis comes from studies that report alleviation of depressive symptoms by using glutamatergic drugs. In particular, drugs that block glutamatergic neurotransmission, like ketamine and riluzole, have antidepressant properties [11]. The glutamate theory is braced by the neuroplasticity alterations observed in MDD in combination with the strengthened HPA axis and neuroinflammatory process [14]. It is proposed that a chronic bolstered HPA activity would increase glucocorticoids, which are identified as enhancers of glutamate release in the PFC and HPC. Subsequently, the chronic exposure of these brain structures to glutamate leads to BDNF downregulation and synaptic pruning [13]. Moreover, the excess glutamate can cause excitotoxicity and promote microglial cell activation [15]. In line with the glutamate overflow in MDD, it is speculated that SSRI-induced SERT blockage may affect mood via impeding presynaptic glutamate release. Chronic elevated levels of extracellular serotonin might regulate the activity of presynaptic serotonin heteroreceptors [14]. For example, a 5HTR which is located mainly in axon terminals is 5HTR1B, which is expressed mostly in the PFC and HPC. 5HTR1B is a Gi-coupled 5HTR and, as a result, exerts an inhibitory effect upon presynaptic neurotransmitter release [16,17,18].

Despite the fact that both FLX and ESC belong to the same class of antidepressants they have substantial differences. Both drugs share a quite similar binding affinity to SERT, with a Ki value close to 1 nM. ESC is ranked first among SSRIs regarding its selectivity to SERT, while FLX has a more broad binding profile that includes 5HTRs and ion channels [19,20,21]. The presence of several binding partners for FLX sets the possibility that the drug may exert its antidepressant action through multiple mechanisms. In the present study, we used primary rat cortical neurons to investigate the additional roles of FLX in synaptic release. Particular attention was given to studies of protein levels of the main components of the soluble *N*-ethylmaleimide-sensitive factor (NSF attachment protein receptor (SNARE complex and its regulation via presynaptic CaV2.1 (P/Q type) voltage-gated Ca^2+^ channels (VGCCs).

## 2. Results

### 2.1. FLX Reduces Presynaptic Activity

**Figure 1:**
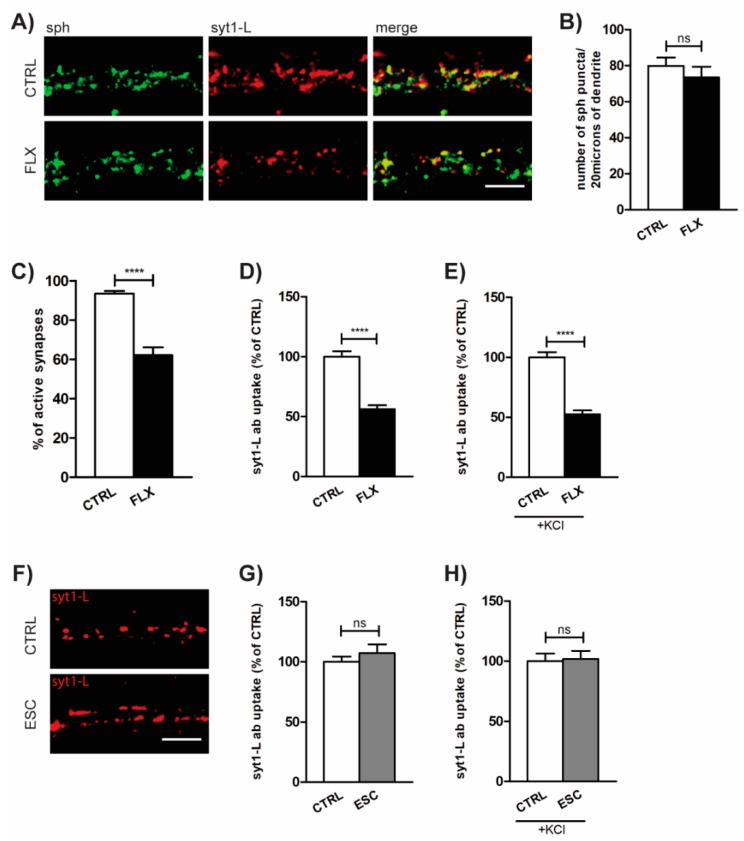
Fluoxetine (FLX) reduces presynaptic activity. (**A**) Representative images of primary cortical neurons treated with FLX (100 µM: 90 min) or control solution (CTRL) and stained with antibody against presynaptic marker synaptophysin (sph; green), syt1-L ab (red), and merged. (**B**) Quantification of the number of sph-positive puncta along 20 µm of proximal dendrite in CTRL (*n* = 29) and FLX-treated cells (*n* = 31). (**C**) Quantification of the percentage of active synapses in CTRL (*n* = 29) and FLX-treated neurons (*n* = 31). Active synapses were determined as puncta positive for both sph and syt1-L ab along 20 µm of proximal dendrite. (**D**) Quantification of “spontaneous” (network-activity-driven) syt1-L ab uptake along 20 µm of proximal dendrite (*n* = 37 CTRL cells; *n* = 39 FLX-treated cells). (**E**) KCl-evoked syt1-L ab uptake along 20 µm of proximal dendrite in CTRL (*n* = 41) and FLX-treated cells (*n* = 41). (**F**) Representative images and (**G**) corresponding quantification of syt1-L ab uptake under “spontaneous” condition (*n* = 35 CTRL cells; *n* = 34 ESC-treated cells) or (**H**) KCL-evoked condition upon the treatment of cells with 100 µM ESC (*n* = 25 cells) or CTRL solution (*n* = 28 cells). In all graphs, bars denote intensity values ± standard error of the mean (SEM) normalized to the mean intensity value in the control group. The statistic was assessed using Mann–Whitney test (**B**,**C**,**G**,**H**) or Student’s *t*-test (**D**,**E**,**G**); **** *p* < 0.0001. Scale bar, 5 µm.

The effect of FLX on presynaptic activity was assessed using synaptotagmin 1 luminal domain antibody (syt1-L ab) uptake assay. This method allows us to measure the rate of both network-activity-driven (“spontaneous”) and depolarization-induced (KCl-evoked) synaptic vesicle (SV) recycling at the level of individual synapses [22]. As shown in Figure 1A–B, treatment of primary cortical neurons with FLX (100 µM; 90 min) did not affect the total number of presynaptic terminals (determined as puncta positive for presynaptic protein synaptophysin (sph)), but significantly reduced the number of active synapses (determined as puncta positive for both sph and syt1-L ab; Figure 1A–C). Also, the total amount of syt1-L ab taken up under both “spontaneous” and KCl-evoked conditions was significantly lower in FLX-treated cells (Figure 1D–E), suggesting the drug-induced reduction of presynaptic activity. In contrast to FLX, the application of the highly SERT-selective SSRI, ESC (100 µM; 90 min), did not have any effect on synaptic function (Figure 1F–H).

**Figure 2:**
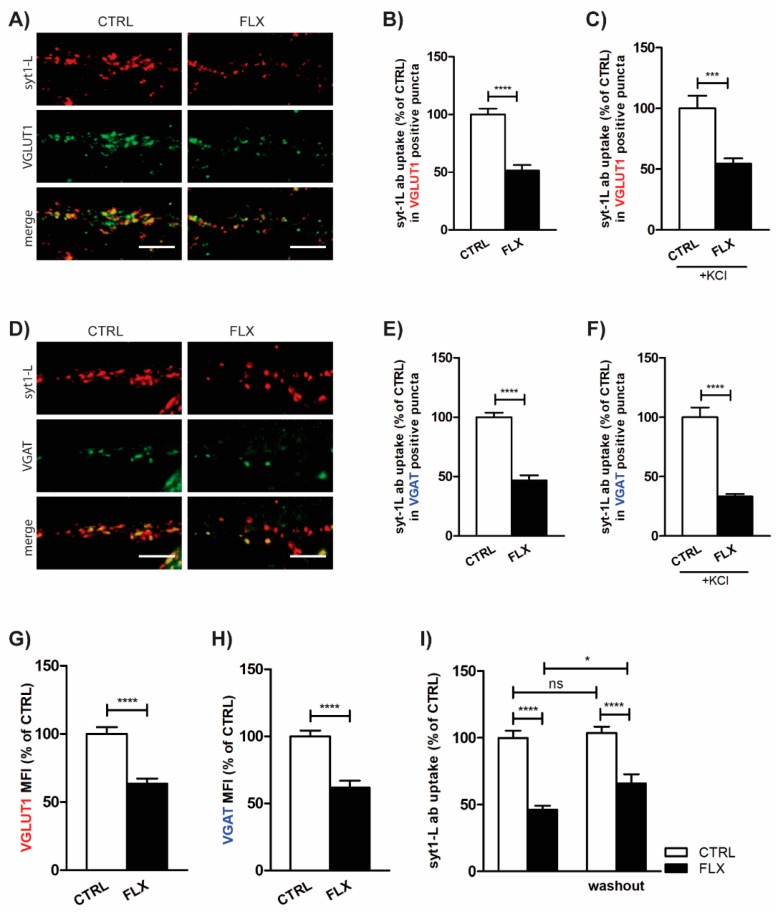
FLX decreases both glutamatergic and GABAergic neurotransmission. (**A**) Representative images of cortical neurons incubated with FLX (100 µM: 90 min) or control solution and stained with syt1-L ab (red) and marker for excitatory synapses (vesicular glutamate transporter 1, VGLUT1, green). Statistical quantification of the intensity of syt1-L ab uptake in puncta positive for VGLUT1 driven by both spontaneous (**B**) and KCl-evoked network activity (**C**) (*n* = 25 CTRL cells; *n* = 23 FLX-treated cells). (**D**) Representative images of cortical neurons incubated with FLX (100 µM: 90 min) or control solution and stained with syt1-L ab (red) and marker for inhibitory synapses (vesicular GABA transporter, VGAT, green). Statistical quantification of the intensity of syt1-L ab uptake in puncta positive for VGAT driven by spontaneous (**E**) and KCl-evoked network activity (**F**) (*n* = 29 CTRL cells; *n* = 24 FLX-treated cells). (**G**) Quantification of VGLUT1 immunoreactivity in CTRL (*n* = 35) and FLX-treated cells (*n* = 33) along 20 µm of proximal dendrite. (**H**) Quantification of VGAT immunoreactivity in CTRL (*n* = 41) and FLX-treated cells (*n* = 31) along 20 µm of proximal dendrite. (**I**) Quantification of syt1-L ab uptake along 20 µm of proximal dendrite 24 h after FLX washout (*n* = 29 CTRL; *n* = 23 FLX; *n* = 31 CTRL/wo; *n* = 24 FLX/wo). In all graphs, values are expressed as means ± SEM and normalized to the mean intensity value in the control group. Statistical significance was assessed by Student’s *t*-test (**B**,**E**,**F**), Mann–Whitney test (**C**,**G**,**H**), and two-way ANOVA followed by Bonferroni multiple comparison test (**I**); ** *p* < 0.01, *** *p* < 0.001 **** *p* < 0.0001. Scale bar, 5 µm.

A previous in vitro study on rat cerebrocortical synaptosomes showed that FLX depresses glutamate release [23]. In our model system, employing syt1-L ab uptake, we were able to measure the activity of both excitatory and inhibitory synapses. These subsets of synapses were determined by co-staining with their specific markers—VGLUT1 (vesicular glutamate transporter 1) for excitatory and VGAT (vesicular GABA transporter) for inhibitory synapses. As shown in Figure 2, FLX application for 90 min equally reduced the level of syt1-L ab uptake in both VGLUT1 (Figure 2A–C) and VGAT positive puncta (Figure 2D–F), indicating its impact on glutamatergic and GABAergic neurotransmission. Moreover, FLX treatment of primary cortical neurons also reduced the total amount of glutamate and GABA transporters at individual synapses (Figure 2G–H), which may account for the reduction of the quantal release per SV.

To investigate whether the inhibitory action of FLX is only transient or has a long-lasting effect, the cells were treated with FLX for 90 min, but presynaptic activity was measured 24 h later. As seen in Figure 2I, washing out the drug for 24 h did not rescue its effect on SV recycling.

### 2.2. FLX Treatment Impairs Exocytic Machinery Components

**Figure 3:**
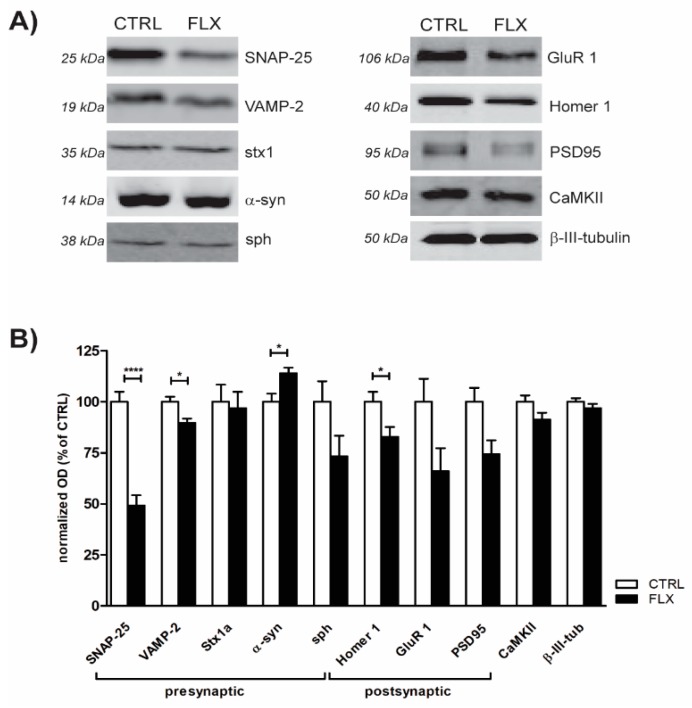
FLX treatment changes the abundance of synaptic proteins. (**A**) Representative immunoblots of selected pre- and postsynaptic proteins isolated from three-week-old cortical neurons treated for 90 min with control solution or 100 µM FLX. (**B**) Quantification of (**A**). The intensity of the signal for each protein was normalized to b-III-tubulin and expressed as a percentage of control. Data originate from ≥3 independent experiments. Statistical significance was evaluated using Student’s *t*-test.

Impaired SV recycling upon FLX treatment may imply the effect of the drug on proteins involved in the regulation of neurotransmitter release. In order to test this, we performed quantitative Western blot to measure the expression of components forming the SNARE core complex (Syntaxin-1 (stx1), SNAP-25, and VAMP-2) and several other pre- and postsynaptic proteins. As shown in Figure 3, application of FLX to primary neurons for 90 min most strikingly attenuated the expression level of SNARE proteins SNAP-25 and VAMP-2, as well as the level of postsynaptic scaffolding protein Homer1. Notably, FLX treatment also lowered (although not to the significant level) the amount of AMPA subunit GluR1 and postsynaptic density protein PSD95 which is essential for anchoring the AMPA receptors to the synapse [24]. In contrast to that, treatment of primary neurons with FLX significantly upregulated the level of α-synuclein, a protein which is highly implicated in several neurodegenerative disorders, including Parkinson’s disease. Altogether, obtained data suggest that FLX treatment changed the abundance of synaptic proteins (primarily SNARE core complex) which, in turn, may alter synaptic function.

### 2.3. Inhibition of P/Q Type Ca2^+^ Channels Mediates FLX-Induced Suppression of Presynaptic Activity

**Figure 4:**
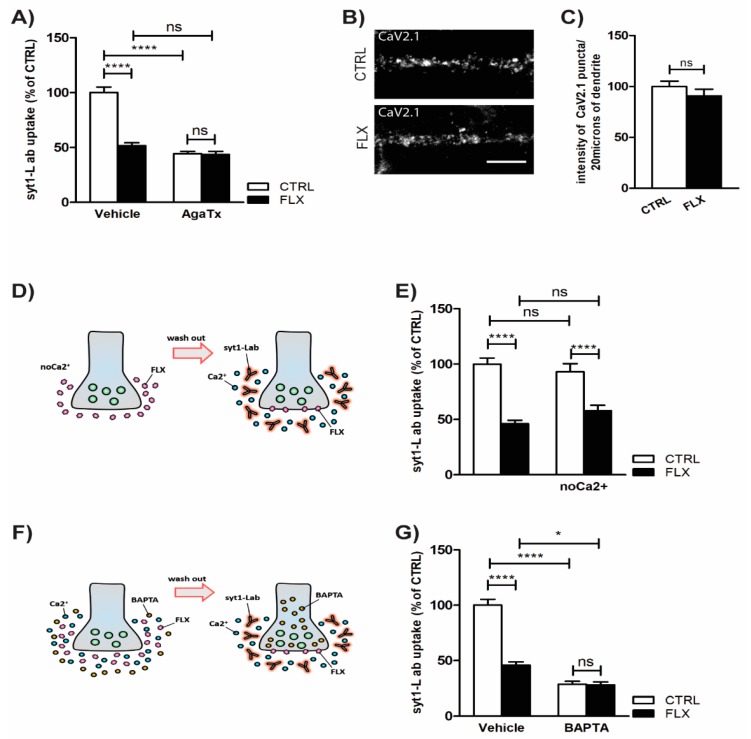
Inhibition of P/Q type Ca^2+^ channels mediates FLX-induced suppression of presynaptic activity. (**A**) Statistical analysis of KCl-evoked syt1-L ab uptake from cortical neurons treated with CTRL solution or FLX (100 µM: 90 min) in the presence or absence of CaV2.1 channels antagonist (AgaTx; 0.4 µM) (*n* = 25 CTRL cells; *n* = 20 FLX; *n* = 28 AgaTx; *n* = 25 AgaTx/FLX). (**B**) Representative images of CTRL and FLX-treated cells stained with antibody against CaV2.1. (**C**) Quantification of (**B**) (*n* = 31 CTRL cells; *n* = 32 FLX-treated cells). (**D**) Schematic description and quantification (**E**) of KCl-evoked syt1-L ab uptake along 20 µm of proximal dendrite in cells treated for 90 min with CTRL solution or 100 µM FLX kept during the treatment in normal medium or Ca^2+^-free medium (*n* = 29 CTRL; *n* = 23 FLX; *n* = 29 no Ca^2+^; *n* = 29 no Ca^2+^/FLX). (**F**) Schematic description and statistical analysis (G) of KCl-driven syt1-L ab uptake from control (*n* = 29) and FLX-treated cortical neurons (*n* = 24) upon chelation of intracellular calcium by BAPTA-AM (10 µM) (*n* = 22 BAPTA-AM; *n* = 15 BAPTA-AM/FLX). In all graphs, values are expressed as means ± SEM. Statistic was done using two-way ANOVA followed by Bonferroni multiple comparison test (**A**,**E**,**G**) or Student’s *t*-test (**C**); * *p* < 0.05, *** *p* < 0.001, **** *p* < 0.0001. Scale bar, 5 µm.

Ca^2+^ influx through VGCCs and subsequent SV fusion are crucial for efficient neurotransmission [25]. Some previous studies already suggested that FLX may inhibit VGCCs in cultured hippocampal cells [26] or cerebrocortical synaptosomes [23]. In our model system, we showed that FLX-induced suppression of SV recycling was completely abolished upon pretreatment of cells with a P/Q type VGCC blocker, AgaTx (Figure 4A). However, when we measured the amount of Cav2.1-positive puncta (P/Q type VGCCs) per synapse, we found no difference between CTRL and FLX-treated cells (Figure 4B–C), further confirming that FLX indeed acts as an efficient blocker of VGCC function. Pretreatment with FLX in a Ca^2+^-free medium did not alter the drug’s effect in the subsequent syt1-L ab uptake (Figure 4D–E), whereas the application of FLX potentiated the effect of membrane-permeable Ca^2+^ chelator, BAPTA-AM. Specifically, preincubation of primary cortical neurons with BAPTA-AM impaired SV release probability, but its inhibitory impact on presynaptic activity was further increased in the presence of FLX (Figure 4F–G).

### 2.4. Impaired Protein Kinase C (PKC) Activity May Account for FLX-Induced Aberrant Neurotransmission

**Figure 5:**
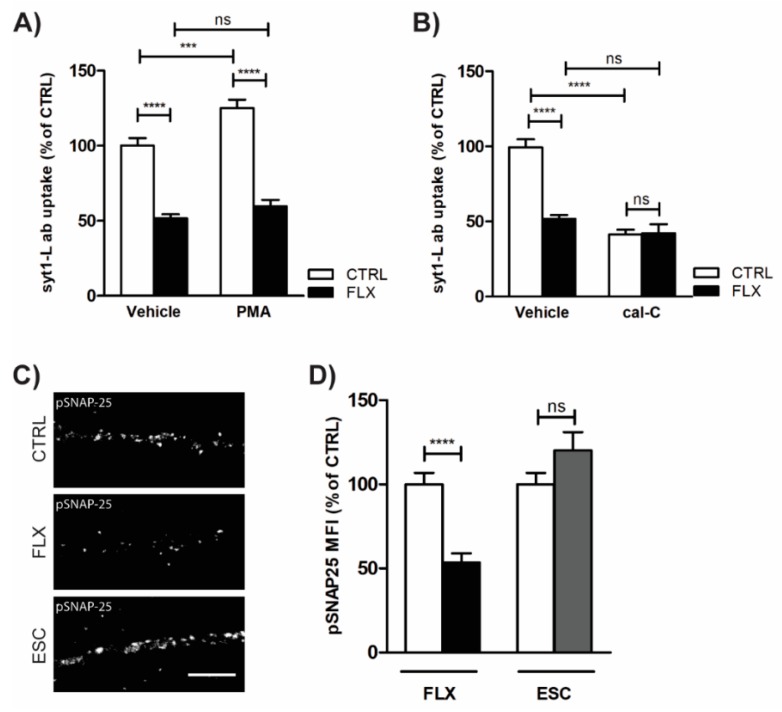
Impaired PKC activity may account for FLX-induced aberrant neurotransmission. (**A**)—Quantification of Syt1-L ab uptake in primary cortical neurons treated with control solution (*n* = 25), protein kinase C (PKC) activator (200 nM PMA; *n* = 29), PMA/FLX (*n* = 25), or FLX (100 µM, 90 min; *n* = 20) alone. (**B**) Quantification of syt1-L ab uptake in primary cortical neurons treated with control solution (*n* = 24), PKC inhibitor (1 µM cal-C; *n* = 31), cal-C/FLX (*n* = 31), or FLX (100 µM, 90 min; *n* = 20) alone. (**C**) Representative images of CTRL, FLX-treated (100 µM, 90 min), and ESC-treated (100 µM, 90 min) neurons stained with antibody against phosphorylated SNAP-25 (Ser 187). (**D**) Quantification of the pSNAP-25 immunofluorescence signal along 20 µm of proximal dendrite in given conditions (*n* = 26 CTRL; *n* = 30 FLX; *n* = 27 ESC). In all graphs, values are expressed as means ± SEM. Statistical significance was assessed using two-way ANOVA followed by Bonferroni multiple comparison test (**A**,**B**) or Mann–Whitney test (**D**); * *p* < 0.05, ** *p* < 0.01, **** *p* < 0.0001. Scale bar, 5 µm.

Altered Ca2^+^ signaling upon FLX treatment has an impact on PKC activity and consequently on PKC-mediated SV fusion. As shown in Figure 5A, FLX profoundly ameliorated PMA-induced presynaptic potentiation, whereas blockade of PKC activity by cal-C completely occluded FLX action on SV recycling (Figure 5B). Together, these data suggested that FLX may impair PKC activity. To further test this assumption, we quantified the level of PKC-mediated phosphorylation of SNAP-25 (pSer 187) in cells exposed to FLX or ESC for 90 min. Our immunofluorescence results clearly demonstrated that FLX but not ESC induces suppression of PKC activity (Figure 5C–D).

## 3. Discussion

### 3.1. FLX Decreases Synaptic Activity through a SERT-Independent Mechanism

In the present study, we report that FLX displays a robust reduction of synaptic vesicle release without affecting the total number of synapses. An obvious explanation of FLX’s ability to decrease neurotransmitter release would be indirectly via the blockade of SERT. However, we showed that FLX is able to reduce synaptic release in primary cortical neurons in culture, which are by definition devoid of serotonin terminals. SSRIs exert their actions through the inhibition of SERT serotonin uptake that is released by the dendrites and axon terminals of neurons located in brainstem raphe [6,27]. PFC and HPC serotonin fibers emerge from dorsal raphe (DR) neurons located in the midline of midbrain [28]. Tryptophan hydroxylase 2 (TPH2) is the enzyme responsible for the production of brain-derived serotonin and is mostly expressed by neurons located in brainstem raphe nuclei [10]. During primary cortical culture preparation, the cortex is detached from midbrain and, as a result, there is no serotonin production within the environment of the culture [29]. However, there is still a possibility that FLX’s effect is a serotonin-independent outcome of SERT blockade. It was shown that SERT is transiently expressed during early postnatal days in the cortical layer VI, layer II of retrosplenial cortex, and CA3 of HPC [30,31]. Accordingly, the primary cortical neurons may express SERT molecules that could be targeted by the FLX applied to the cell culture media. To check this possibility, we also measured the presynaptic activity upon application of the highly selective SERT blocker, ESC. However, in contrast to FLX, ESC did not alter the syt1-L ab uptake signal. Taken together, we concluded that SERT blockade is not the mechanism underlying the changes in synaptic activity exerted by FLX.

### 3.2. FLX Decreases the Level of Synaptic Machinery Proteins, Especially Core SNARE Molecules

In order to investigate the synaptic mechanism of FLX-induced reduction in vesicular release, we performed Western blot (WB) analysis of proteins crucial for the proper function of synapses. It was published previously that chronic administration of FLX in mice lowered the levels of SNARE complex proteins SNAP-25 and vesicle-associated membrane protein (VAMP2) based on their HPC synaptic membrane fraction [32]. Similarly, in our study, we showed that even short-term treatment with FLX also downregulated SNAP-25, VAMP-2, and postsynaptic scaffolding protein Homer1. Nevertheless, the strongest effect was observed in SNAP-25, as the drug application dropped the protein level to almost 50%. Therefore, FLX may impair SNARE complex formation and function, which would subsequently weaken synaptic vesicle release probability. FLX might decrease SNAP-25 either through interrupting its transcriptional/translational process or enhancing its degradation. A crucial regulator of SNAP-25 misfolding and subsequent degradation is cysteine string protein α (CSPα). Broadly, CSPα works as a SNAP-25 chaperone and prevents its ubiquitination during SNARE-complex assembly [33]. There is a possibility that FLX hinders the ability of CSPα to interact with SNAP-25 and leads to its downregulation that we observed. Interestingly, CSPα knock-out mice display low amounts of SNAP-25 but also remarkable signs of neurodegeneration [34,35]. It was found that an assisting protein in CSPα function is α-synuclein which is critically implicated in the neurodegenerative process of PD [34,36]. In our study, we showed that FLX leads to a significant upregulation of α-synuclein. 

### 3.3. FLX Inhibits Ca^2+^-Induced Activation of PKC through Blocking the P/Q Type VGCCs

In order to explore further the observed reduction of presynaptic activity and altered SNARE complex, we investigated the role of PKC in FLX’s action. PKC is known to be crucial effector of synaptic activity as its phosphorylation targets play a critical role in vesicular docking. The phosphorylation targets of PKC include CSPα and SNAP-25 [37,38]. Our data clearly showed that FLX but not ESC reduced the level of SNAP-25 at Ser 187, which is known to be phosphorylated by PKC [39]. Furthermore, we also showed that FLX blocks the ability of PMA to enhance synaptic vesicle fusion, whereas co-application of PKC inhibitor calphostin-C and FLX had no synergistic effect, signifying a potential link between the antidepressant and the kinase activity [40]. PKC activation requires the binding of three factors: diacylglycerol to C1 domain, Ca^2+^ to C2 domain, and ATP to C3 domain [41]; thus, we suppose that FLX may affect one of those factors. Application of intracellular Ca^2+^ chelators to the primary culture enhanced FLX’s ability to reduce synaptic activity, suggesting a possible dependency of the drug’s action on Ca^2+^ influx. Furthermore, by adding agatoxin (AgaTx) to the medium, we showed that FLX may act as a P/Q type VGCC blocker. Supporting our findings, several calcium imaging studies showed that FLX decreases intracellular Ca^2+^ [42,43,44]. Decisively, we propose that FLX blocks P/Q type VGCC channels, which would decrease Ca^2+^-induced activation of PKC and lead to subsequent reduction of SNARE and vesicular fusion. However there is also an evidence that selective deletion of SNAP-25 from the synaptic formation does not affect dramatically the synaptic function [34], so the observed fluoxetine-induced diminish of synaptic efficiency may also be explained by the simultaneous loss of VAMP2 and/or by the net loss of function of the rest of presynaptic PKC targets. 

Our hypothesis that FLX acts as a P/Q type VGCC blocker is supported by the study of Wang et al. [23]. However, this study was restricted to the effect of FLX on glutamate release, while we described here a more generalized effect of the drug in both glutamatergic and GABAergic neurons. Notably, suppressed activity of PFC GABAergic interneurons was interlinked with anxiety and depression pathophysiology [45]. For example, it was shown that selective activation of somatostatin-positive GABAergic interneurons induce anxiolytic and less depressive behavior. As a result, the possible inhibition of these neurons by FLX may counteract its antidepressant and anxiolytic properties [46,47,48]. Over the last few years, there was growing preference in prescribing ESC instead of FLX, due to its higher tolerability and efficacy [49]. There is a possibility that FLX’s efficacy and tolerability as an SSRI may be affected by its ability to inhibit GABAergic neurotransmission. We propose that FLX’s P/Q VGCC blockade reduces SNAP-25 levels, which in turn hampers GABA release by forebrain interneurons. Interestingly, this effect is supported by studies that show low post-mortem levels of forebrain SNAP-25 in several neuropsychiatric disorders like bipolar disorder, schizophrenia and MDD [50,51,52]. Therefore, FLX may negatively affect the course of MDD due to its ability to decrease SNAP-25. Moreover, in the present study, we introduced a possible binary role of FLX in the neurodegenerative process. Due to the calcium overload hypothesis, P/Q type VGCCs were used as a pharmaceutical target for neurodegenerative diseases [53,54,55]. According to this previous fact, FLX may have beneficial effects in neuronal survival. However, we showed that FLX’s P/Q type blockade induced a simultaneous reduction and upregulation in SNAP-25 and α-synuclein, respectively. The low levels of SNAP-25 are correlated with signs of PD-like neurodegeneration [34]. Furthermore, it is known that α-synuclein overexpression prevents SNAP-25 degradation and rescues the neurodegenerative phenotype [36,37,38,39]. This effect could explain the reactive upregulation of α-synuclein that we observed in our study.

**Figure 6:**
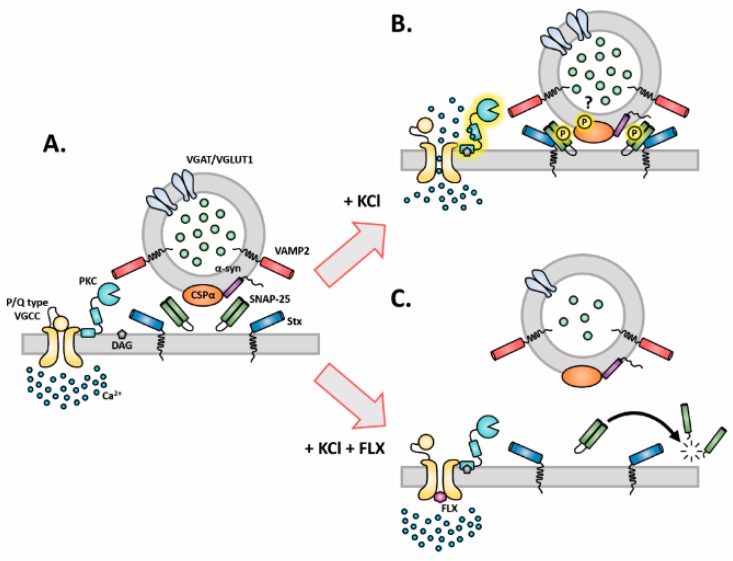
Schematic representation of FLX’s effects on presynapse. (**A**) The presynaptic membrane and synaptic vesicles at rest. (**B**) The activation of PKC upon KCl stimulation and its possible effects on synaptic machinery proteins such as cysteine string protein alpha (CSPα) and SNAP-25. (**C**) The impact of FLX on SNAP-25 through PKC. FLX: fluoxetine, CSPα: cysteine string protein alpha, α-syn: alpha-synuclein, SNAP-25: synaptosomal nerve-associated protein 25, PKC: protein kinase C, Stx: syntaxin, VAMP2: vesicle-associated membrane protein 2, VLUT1: vesicular glutamate transporter 1, VGAT: vesicular GABA transporter, DAG: diacyglycerol, VGCC: voltage-gated calcium channel, Ca^2+^: calcium.

Taken together, we report that the FLX-induced decrease in vesicular exocytosis may be explained through the diminished protein levels of main components of the SNARE core complex (SNAP-25 and VAMP-2). Furthermore, we propose that these changes are the consequence of interrupted protein kinase C (PKC) activation by calcium (Ca^2+^) influx. This interruption occurs via direct inhibition of presynaptic P/Q type VGCCs by FLX (Figure 6). Conclusively, we propose that FLX blocks presynaptic calcium influx, which hinders the normal function of the soluble SNARE complex, through SNAP-25 and VAMP-2 reduction. These data shed new light on the mechanism(s) of action of FLX, which could have implications for the role of FLX in MDD, anxiety, neurodegenerative diseases, and stroke.

## 4. Materials and Methods

### 4.1. Animals

For the preparation of primary cortical neurons, we used time pregnant Wistar rats from Charles River (Sulzfeld, Germany). All animal work was performed in agreement with the European Council Directive (86/609/EEC) and approved by the local Animal Ethics Committee (Stockholms Norra Djurförsöksetiska Nämnd, approval number N270/15, 03 December 2015.).

### 4.2. Chemical Reagents

Fluoxetine HCl (FLX, 100 µM, LKT Laboratories, St. Paul, MN, USA), escitalopram oxalate (ESC, 100 µM, BioTrend, Miramar Beach, FL, USA), ω-agatoxin TK (AgaTx, 0.4 µM, Tocris, Bristol, UK), BAPTA-AM (10 µM, Tocris, Bristol, UK), phorbol 12,13-dibutyrate (PMA, 200 nM, Tocris, Bristol, UK), and calphostin C (1 µM, Tocris, Bristol, UK) were used. In all experiments, cells were treated with FLX or ESC for 90 min, and other drugs were added 20 min before FLX application.

### 4.3. Preparation of Primary Neurons

Primary cortical neurons were essentially prepared as described previously [22]. Briefly, rat brains from embryonic day 18 (E18) were dissociated with 0.25% trypsin for 20 min and plated at the desired density in Dulbecco’s Modified Eagle Medium (DMEM) containing 10% fetal bovine serum (FBS), antibiotics (100 U/mL penicillin, 100 µg/mL streptomycin), and 0.8 mM glutamine. Then, 24 h after the plating, DMEM was replaced with neurobasal medium supplemented with B27, antibiotics, and 0.8 mM glutamine. All cells were kept in a humidified incubator with 5% CO_2_ up to three weeks. For immunocytochemistry, cells were plated on poly-d-lysine-coated glass coverslips (12 mm) at a density of 50,000 cells/coverslip in 24-well plates. For biochemical experiments, cells were plated in six-well plates at a density of 300,000 cells/well.

### 4.4. Immunocytochemistry and Syt1-L ab Uptake Assay

After the corresponding treatment, primary cortical neurons were fixed for 3 min with 4% paraformaldehyde in phosphate-buffered saline (PBS), blocked and permeabilized with PBS containing 10% FBS, 0.1% glycine, and 0.3% TritonX-100 for 30 min, and stained with primary antibodies overnight at 4 °C. On the next day, cells were washed three times with PBS and incubated for 1 h at room temperature with fluorescently labeled secondary antibodies. Both primary and secondary antibodies were diluted in PBS containing 3% FBS. After the secondary antibodies, coverslips were washed three times with PBS and maintained on microscopic slides with Mowiol (Calbiochem). Synaptotagmin-1 luminal domain antibody uptake (syt1-L ab uptake) was essentially done as described previously [22]. After the corresponding treatment, cells were washed two times with freshly prepared Tyrode’s buffer (containing 119 mM NaCl, 2.5 mM KCl, 2 mM CaCl_2_, 2 mM MgCl_2_, 30 mM glucose, and 25 mm hepes (4-(2-hydroxyethyl)-1-piperazineethanesulfonic acid); pH 7.4) and subsequently incubated with fluorescently-labeled syt1-L antibody diluted in the same buffer. To assess network-activity-driven syt1-L ab uptake, the incubation was 20 min at 37 °C, and, to assess KCl-evoked syt1-L ab uptake, the incubation was done for 4 min in 50 mM KCl–Tyrode’s buffer. Thereafter, samples were fixed and stained as described above. In each experiment, at least two coverslips per treatment were processed in parallel. Data originated from ≥3 independent experiments.

### 4.5. Western Blot

Sample preparation and conditions for Western blot (WB) were as described previously [56]. In brief, three-week-old primary cortical cells were treated as indicated, washed briefly with ice-cold washing buffer (10 mM Tris, 300 mM sucrose pH 7.4), and lysed in a buffer containing 10 mM Tris-HCl (pH 7.4), 150 mM NaCl, 2% sodium dodecyl sulfate (SDS), 1% deoxycholate, and 1% TritonX-100, supplemented with protease and phosphatase inhibitors. Lysates were centrifuged for 10 min at 2000× *g* to eliminate cell debris. Protein concentration was determined by the colorimetric BCA (bicinchoninic acid) Protein Assay (Pierce), and equal amounts of proteins were loaded onto a polyacrylamide gel. Primary antibodies were applied overnight at 4 °C and fluorescently labeled secondary antibodies for 1 h at room temperature (RT). For immunoblotting with α-synuclein, before incubation with primary antibody, the membrane was fixed for 30 min with 4% paraformaldehyde (PFA). Membranes were scanned using an Odyssey CLx Infra Red Imaging system from LI-COR Biosciences (Lincoln, NE, USA). Quantification of the signals was done using software Image Studio 3.1.

### 4.6. Antibodies

Primary polyclonal antibodies from rabbit were as follows: syntaxin-1 (WB 1:1000, Synaptic Systems, Göttingen, Germany); α-Synuclin (C-20)-R (WB 1:1000; Santa Cruz, Dallas, TX, USA); synaptophysin (WB 1:1000, Sigma, St. Louise, MO, USA); GluR1 (WB 1:1000, Upstate Biotechnology, Lake Placid, NY, USA); Homer 1 (WB 1:1000, Synaptic Systems, Göttingen, Germany); CaMKII (WB 1:1000; Cell Signaling, Danvers, MA, USA); CaV2.1 (ICC 1.500; Alomone labs, Jerusalem, Israel); VGAT (ICC 1:500, Synaptic Systems, Göttingen, Germany); VGLUT1 (ICC 1:500, Synaptic Systems, Göttingen, Germany); phospho-SNAP25 (Ser187) (ICC 1:500; Thermo Fischer, Waltham, MA, USA). Primary monoclonal antibodies from mouse were as follows: Synaptotagmin-1 lumenal domain fluorescence labeled with Oyster^®^ 550 (ICC 1:250 Synaptic Systems, Göttingen, Germany); SNAP-25 (WB 1:1000, Synaptic Systems, Göttingen, Germany); Synaptobrevin-2/VAMP-2 (WB 1:1000; Synaptic Systems, Göttingen, Germany); PSD95 (WB 1:1000; Synaptic Systems, Göttingen, Germany); beta-III-tubulin (WB 1:2000, Sigma, St. Louise, MO, USA). Primary polyclonal antibodies from guinea pig were as follows: Synaptophysin-1 (ICC 1:500 Synaptic Systems, Göttingen, Germany). Fluorescently labeled secondary antibodies used for immunocytochemistry (ICC) were purchased from Thermo Scientific and used in a dilution of 1:1000 (anti-rabbit Alexa Fluor^®^ 488 conjugate to anti-guinea pig Alexa Fluor^®^ 647 conjugate). Fluorescently labeled secondary antibodies used for WB were obtained from Li-COR: anti-rabbit IRDye 800CW (1:30.000) and anti-mouse IRDye 680RD (1:30.000).

### 4.7. Image Acquisition and Analysis

Images were taken with a ZEISS LSM 880 Airyscan confocal laser scanning microscope equipped with ZEN2.1 software, using a Plan-Apochromat 63×/1.4 Oil DIC M27 63× oil objective. Preparation of images and quantification of the immunofluorescence (IF) was done using Image J (National Institutes of Health, NIH, http://rsb.info.nih.gov/ij/) and OpenView software [57]. Firstly, the background was subtracted from all channels using Image J, and the synaptic IF intensities were assessed in a region of interest (along 20 µm of the proximal dendrite) which was set by the mask in the channel for synaptophysin. This mask was created semi-automatically by using OpenView. The final adjustment of images was made by ImageJ and Photoshop (Adobe Systems, San Jose, CA, USA).

### 4.8. Statistics

Statistical analyses were done using GraphPad Prism, version 5.04. The normal distribution of the data was assessed by a D’Agostino–Pearson normality test and, accordingly, parametric or nonparametric tests were applied. For all ICC analysis, data within one experimental set-up were normalized to the mean of the control group and expressed as means ± standard error of the mean (SEM). Final data were obtained from ≥2 independent cell culture preparations (each with ≥2 independently processed coverslips/treatments). The number (*n*) stated in each figure legend refers to the number of analyzed cells derived from those independent experimental set-ups. For qWB, all values were first normalized to beta-III-tubulin referred as a loading control for each membrane. These normalized values of the treated groups were further expressed as percentages of the control group for each experiment. The final data originated from 3–7 independent experiments pulled together. Statistical significance was assessed as follows: * *p* < 0.05; ** *p* < 0.01; *** *p* < 0.001; **** *p* < 0.0001.

## Abbreviations

SSRIs	selective serotonin reuptake inhibitors
SERT	serotonin transporter
FLX	fluoxetine
GABA	gamma-Aminobutyric acid
SNAP-25	synaptosomal nerve-associated protein 25
PKC	protein kinase C
VGCCs	voltage-gated calcium channels
NSF	*N*-ethylmaleimide-sensitive factor
SNARE	soluble NSF attachment protein receptor
MDD	major depressive disorder
HPA	hypothalamic–pituitary axis
ESC	escitalopram
5HTR	5-hydroxytryptamine receptor
BDNF	brain-derived neurotrophic factor
PFC	prefrontal cortex
HPC	hippocampal complex
SV	synaptic vesicle
Sph	synaptophysin
syt1-L ab	synaptotagmin 1 luminal domain antibody
VGLUT1	vesicular glutamate transporter 1
VGAT	vesicular GABA transporter
VAMP-2	Vesicle-associated membrane protein 2
AMPA	α-amino-3-hydroxy-5-methyl-4- isoxazole-propanoic acid
PSD95	postsynaptic density protein 95
BAPTA-AM	1,2-Bis(2-aminophenoxy)ethane-N,N,N′,N′-tetraacetic acid tetrakis(acetoxymethyl ester)
PMA	phorbol myristate acetate
cal-C	Calphostin C
SNAP-25	Synaptosomal nerve-associated protein 25
DR	dorsal raphe
TPH2	Tryptophan hydroxylase 2
CSPα	cysteine string protein α
PD	Parkinson’s disease
